# Establishment of a nomogram model to predict macrosomia in pregnant women with gestational diabetes mellitus

**DOI:** 10.1186/s12884-021-04049-0

**Published:** 2021-08-22

**Authors:** Yujiao Zou, Yan Zhang, Zhenhua Yin, Lili Wei, Bohan Lv, Yili Wu

**Affiliations:** 1 School of Nursing, Qingdao University, Qingdao, China; 2 Nursing Department, Affiliated Hospital of Qingdao University, Qingdao, China; 3 School of Public Health, Qingdao University, Qingdao, China

**Keywords:** Macrosomia, Gestational diabetes mellitus, Nomogram, Prediction model

## Abstract

**Aim:**

To establish a nomogram model to predict the risk of macrosomia in pregnant women with gestational diabetes mellitus in China.

**Methods:**

We retrospectively collected the medical records of 783 pregnant women with gestational diabetes who underwent prenatal examinations and delivered at the Affiliated Hospital of Qingdao University from October 2019 to October 2020. The pregnant women were randomly divided into two groups in a 4:1 ratio to generate and validate the model. The independent risk factors for macrosomia in pregnant women with gestational diabetes mellitus were analyzed by multivariate logistic regression, and the nomogram model to predict the risk of macrosomia in pregnant women with gestational diabetes mellitus was established and verified by R software.

**Results:**

Logistic regression analysis showed that prepregnancy body mass index, weight gain during pregnancy, fasting plasma glucose, triglycerides, biparietal diameter and amniotic fluid index were independent risk factors for macrosomia (*P* < 0.05). The areas under the ROC curve for internal and external validation of the model were 0.813 (95 % confidence interval 0.754–0.862) and 0.903 (95 % confidence interval 0.588–0.967), respectively. The calibration curve was a straight line with a slope close to 1.

**Conclusions:**

In this study, we constructed a nomogram model to predict the risk of macrosomia in pregnant women with gestational diabetes mellitus. The model has good discrimination and calibration abilities, which can help clinical healthcare staff accurately predict macrosomia in pregnant women with gestational diabetes mellitus.

## Background

Gestational diabetes mellitus (GDM) is one of the most common endocrine and metabolic diseases in pregnancy, and is a form of diabetes mellitus that occurs and is first discovered during pregnancy [1]. GDM is also one of the main risk factors for macrosomia [2]. Previous studies have shown that the incidence of macrosomia among pregnant women with GDM is more than one time higher than that among normal pregnant women [3, 4]. In recent years, with the standardization of pregnancy care, the overall incidence of macrosomia has decreased [5], but pregnant women with GDM are still at high risk for macrosomia, with an incidence greater than 15 % in some areas [6, 7]. Macrosomia is defined as a newborn with birth weight ≥ 4000 g [8]. It is not only associated with shoulder dystocia, postpartum hemorrhage and other adverse pregnancy outcomes [9, 10] but also increases the incidence of neonatal hypoglycemia and neonatal jaundice [3]. Macrosomia has even been reported to affect the long-term physical and mental health of offspring [11, 12], placing a large financial and psychological burden on mothers and babies. Vinter [13] conducted a retrospective cohort study of 3,098 mothers of macrosomia who delivered between 2000 and 2015, and the results showed that women with a prenatal prediction of macrosomia had a significantly reduced risk of adverse pregnancy outcomes compared with women with unpredicted macrosomia. Therefore, accurate prenatal prediction of macrosomia can help improve maternal and infant outcomes and ensure maternal and infant health.

Currently, the most commonly used method for the clinical prediction of macrosomia is the Hadlock ultrasonic formula built into the ultrasonic instrument. This formula was established by Hadlock in the 1980 s using Western populations as a sample, and there are certain deviations when applied to Chinese populations [14, 15]. In addition, some scholars believe that GDM can lead to excessive accumulation of fat in the fetus, and ultrasound for fetal weight prediction depends mainly on bone markers. Therefore, relying only on ultrasonic measurement parameters to predict macrosomia in pregnant women with GDM may lead to inaccurate prediction results [15–17]. This study aims to establish a comprehensive, simple, and feasible personalized tool to help accurately predict the risk of macrosomia among pregnant women with GDM in China.

## Methods

### Study design

This is a retrospective study that was approved by the Ethics Committee of the Affiliated Hospital of Qingdao University. Informed consent was waived due to the retrospective nature of the study.

### Participants

We reviewed the medical records of pregnant women with GDM who underwent obstetric examination and delivered at The Affiliated Hospital of Qingdao University from October 2019 to October 2020. Pregnant women with singleton pregnancies who were older than 18 years and diagnosed with GDM were recruited. Those with pregnancy complications (e.g., pregnancy hypertension, anemia, etc.) or pregnancy with other diseases (e.g., pulmonary hypertension) or fetal malformation were excluded. GDM was diagnosed using the diagnostic criteria published by the International Association of Diabetes and Pregnancy Study Groups (IADPSG) in 2010 [18], with the 75 g oral glucose tolerance test (OGTT) being performed in pregnant women at 24–28 weeks of gestation. The thresholds of fasting plasma glucose (FPG), 1 and 2 h after taking glucose were 5.1 mmol/L, 10.0 mmol/L and 8.5 mmol/L, respectively. GDM was diagnosed when any of the above thresholds was reached or exceeded.

### Variables included for analysis

Demographic variables included age, self-reported prepregnancy weight and height, weight at the last prenatal examination, number of pregnancies, parity, gestational age at delivery, history of abortion, diabetes in first-degree relatives and whether the menstrual cycle was regular. Prepregnancy body mass index (prepregnancy BMI) was calculated by dividing the prepregnancy weight (kg) by the prepregnancy height (m^2^). Weight gain during pregnancy was calculated by subtracting prepregnancy weight from weight at the last prenatal examination. Laboratory indexes included the results of the 75 g OGTT in the second trimester, triglycerides (TG) and total cholesterol (TC) at 28–32 weeks of gestation, umbilical blood flow (S/D), pulsatility index (PI), resistance index (RI), biparietal diameter (BPD), head circumference (HC), femur length (FL) and amniotic fluid index (AFI) at the last prenatal examination. The occurrence of macrosomia was the primary outcome of this study. Shortly after birth, the newborns were weighed, and the weights were recorded by the medical staff. Those with birth weights ≥ 4000 g were considered to have macrosomia.

### Statistical analysis

In the initial statistical analysis, we evaluated whether the data followed a normal distribution. For continuous variables following a normal distribution, means and standard deviations were used. Classification variables were expressed as counts and percentages. The t-test or rank sum test was used to compare the differences between groups for continuous variables, and the chi-square test was used to compare the differences between groups for classified variables. The initial dataset was randomly divided into a training set and a validation set in a 4:1 ratio to generate and validate the model, respectively. To determine the risk characteristics of pregnant women with GDM who delivered newborns with macrosomia, a multivariate logistic regression analysis was performed using a forward stepwise approach within the training set, and a collinearity test was performed on the logistic regression model. This method included all variables with unified measurements less than 0.05 in univariate analysis. Finally, the regression coefficient of each variable and the odds ratio of the bilateral 95 % confidence interval were calculated. The nomogram model was drawn according to the fitted logistic regression model to predict the occurrence of macrosomia in pregnant women with GDM.

Model validation consisted of two parts: internal and external validation. First, internal validation was performed with a bootstrap process that used 1000 resamples in the training set. Discriminant ability was studied by analyzing the area under the ROC curve. In addition, a calibration curve was drawn to quantify the consistency between the incidence of macrosomia predicted by the nomogram model and the actual incidence of macrosomia. Second, external authentication was performed in a validation group.

## Results

### Baseline characteristics for the two cohorts

A total of 783 pregnant women with GDM were included in this study. Among them, 99 pregnant women gave birth to newborns with macrosomia. The initial data set was divided into a training set (*n* = 626) and a validation set (*n* = 157) at a ratio of 4:1. In the training set, 13.26 % of the pregnant women gave birth to newborns with macrosomia, and in the validation set, 10.19 % of the pregnant women gave birth to newborns with macrosomia. We compared the intergroup differences between the macrosomia group and the nonmacrosomia group in the training set. The results showed that the prepregnancy BMI, weight gain during pregnancy, FPG, TG, BPD and AFI in the macrosomia group were significantly higher than those in the nonmacrosomia group, while the RI and PI were lower than those in the nonmacrosomia group (Table 1).

**Table 1:** Comparison of clinical data between the macrosomia group and the nonmacrosomia group in the training set (*N* = 626)

Variables	Nonmacrosomia *n* = 543	Macrosomia *n* = 83	*P*
Age, years	33.01 ± 4.47	33.11 ± 4.90	0.858
Prepregnancy BMI, kg/m2	23.20 ± 3.52	24.47 ± 3.40	0.002
Number of pregnancies	2.28 ± 1.27	2.23 ± 1.18	0.723
Parity	0.56 ± 0.57	0.54 ± 0.55	0.771
Gestational age at delivery, weeks	38.16 ± 1.72	38.54 ± 1.51	0.057
Weight gain during pregnancy, kg	13.00 ± 4.68	14.70 ± 4.73	0.002
Fasting plasma glucose (FPG), mmol/L	5.03 ± 0.55	5.33 ± 0.77	0.001
1 h 75 g glucose level, mmol/L	9.72 ± 1.62	9.95 ± 3.91	0.594
2 h 75 g glucose level, mmol/L	8.15 ± 1.45	8.01 ± 2.46	0.610
Triglycerides (TG), mmol/L	3.33 ± 1.62	4.02 ± 1.69	< 0.001
Total cholesterol (TC), mmol/L	6.11 ± 1.17	6.15 ± 1.20	0.756
Umbilical artery blood velocity (S/D)	2.19 ± 0.36	2.12 ± 0.32	0.087
Biparietal diameter (BPD), cm	9.26 ± 0.46	9.59 ± 0.42	< 0.001
Resistance index (RI)	0.53 ± 0.07	0.52 ± 0.08	0.035
Pulsatility index (PI)	0.78 ± 0.18	0.73 ± 0.14	0.016
Head circumference (HC), cm	32.79 ± 1.34	33.79 ± 1.67	< 0.001
Femur length (FL), cm	7.06 ± 0.39	7.27 ± 0.40	< 0.001
Amniotic fluid index (AFI), cm	12.51 ± 3.42	14.31 ± 3.92	< 0.001
Whether the menstrual cycle is regular, n (%)			
Yes	479	74	0.803
No	64	9	
Diabetes in first degree relatives, n (%)			
Yes	57	9	0.924
No	486	74	
History of abortion, n (%)			
Yes	266	42	0.784
No	277	41	

### Logistic regression analysis of risk factors for macrosomia

A dichotomous logistic regression model was established with macrosomia as the dependent variable and significant indicators of univariate analysis as the covariates. The results showed that there were 6 independent predictors, including prepregnancy BMI, weight gain during pregnancy, fasting plasma glucose levels in the 75 g OGTT, TG at 28–32 weeks of gestation, BPD and AFI in the third trimester (Table 2). The results of the collinearity test show that the VIF values of all variables are less than 10, which can be preliminarily considered to indicate that the problem of collinearity can be ignored.

**Table 2:** Multivariate logistic regression analysis of macrosomia in pregnant women with gestational diabetes mellitus (*N* = 626)

Variables	B	SE	OR(95 %CI)	*P*
Prepregnancy BMI	0.109	0.035	1.116(1.041–1.195)	0.002
Weight gain during pregnancy	0.092	0.026	1.097(1.041–1.155)	< 0.001
Fasting plasma glucose (FPG)	0.588	0.200	1.800(1.217–2.661)	0.003
Triglycerides (TG)	0.186	0.063	1.204(1.064–1.363)	0.003
Biparietal diameter (BPD)	2.179	0.370	8.839(4.279–18.256)	< 0.001
Amniotic fluid index (AFI)	0.094	0.034	1.099(1.027–1.175)	0.006

### Nomogram and evaluation of prediction model for macrosomia

**Fig. 1:**
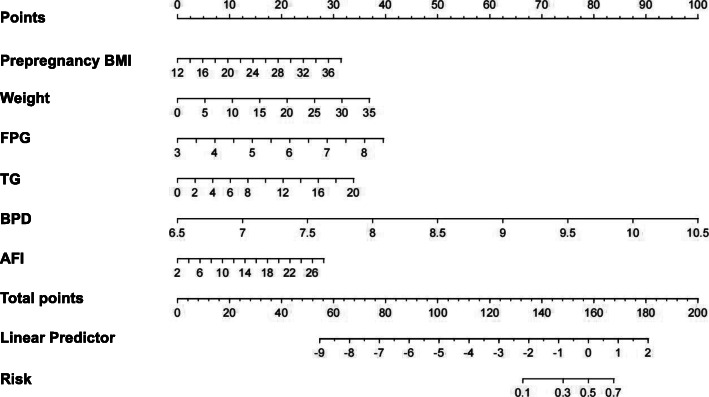
Nomogram model for predicting the risk of macrosomia in pregnant women with gestational diabetes mellitus. BMI, body mass index; weight, weight gain during pregnancy; FPG, fasting plasma glucose; TG, triglycerides; BPD, biparietal diameter; AFI, amniotic fluid index

**Fig. 2:**
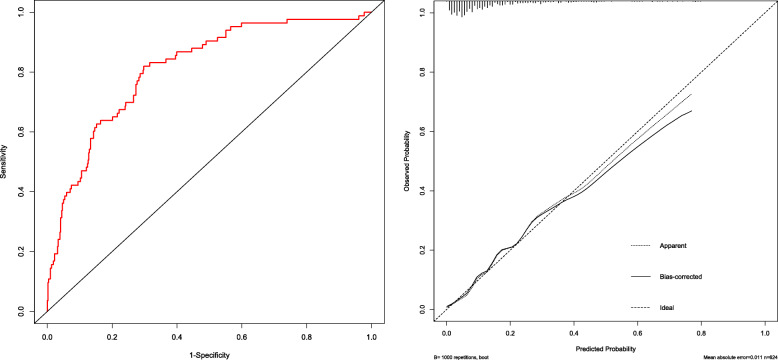
Internal validation of the nomogram model in the training set. The ROC curve of GDM macrosomia is shown on the left, and the calibration curve of the macrosomia curve is shown on the right

**Fig. 3:**
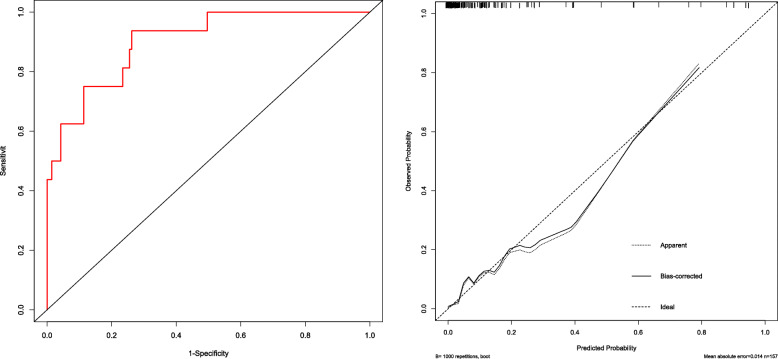
External validation of the nomogram model in the validation set. The ROC curve of GDM macrosomia is shown on the left, and the calibration curve of the macrosomia curve is shown on the right

A nomogram model incorporating prepregnancy BMI, weight gain during pregnancy, FPG and TG was developed and is presented in Fig. 1. A 1000 bootstrap analysis was used to verify the nomogram model. After receiving internal certification, this nomogram model validated a perfect discriminative capacity with an AUC of 0.813 (95 % CI: 0.764–0.862). The standard curve revealed the possibility of using the nomogram model to predict the actual probability of macrosomia in a pregnant woman with GDM (Fig. 2). External authentication was achieved by comparing the predictive nomogram model and individual actual possibility in the authentication group. For the validation group, the AUC of the nomogram model was 0.903 (95 % CI: 0.858–0.967). In addition, the standardized graph showed that the nonparametric curve fit well with the ideal line, indicating that the observed probability was very similar to the predicted probability (Fig. 3). The AUCs were both above 0.8 in the two verifications, indicating that the model had good distinguishing ability.

## Discussion

Our study showed that 99 of the 783 pregnant women with GDM gave birth to newborns with macrosomia, with an incidence of 12.64 %, which was higher than the incidence of macrosomia among normal pregnant women [19]. Gorban investigated 1870 pregnant women with GDM and found that the incidence of macrosomia was 12.9 %, similar to the results of our study [20].

The occurrence of macrosomia is affected by many factors. In this study, the medical records of 783 pregnant women were analyzed retrospectively. Considering the statistical significance and professional significance, 20 easily available indicators were selected for model analysis. The results showed that BMI before pregnancy, weight gain during pregnancy, FPG, TG, BPD and AFI were risk factors for macrosomia in pregnant women with GDM. Our study found that prepregnancy obesity and excessive weight gain during pregnancy may increase the risk of macrosomia, which is consistent with previous studies [21, 22]. The higher the BMI before pregnancy is, the more likely pregnant women are to have poor eating behaviors, such as high-fat and high-sugar diets, or poor living habits, such as a sedentary lifestyle. Under normal circumstances, the weight gain during pregnancy of such pregnant women is also difficult to control [23], suggesting that we should not only strengthen pregnancy management but also pay attention to prepregnancy health guidance. Pregnant women should be instructed to strengthen weight management to improve the impact of weight on the fetus. At present, the recommended value of weight gain during pregnancy issued by the American Institute of Medicine (IOM) in 2009 is widely used in China [24]. However, in recent years, studies have found that, due to the great differences in genetic characteristics, dietary structure, lifestyle and other aspects between the two countries, the recommended value is not completely applicable to Chinese pregnant women [25, 26]. Therefore, we still need to develop a recommended pregnancy weight gain value for Chinese women. Other studies have confirmed that, in addition to FPG, increased postprandial blood glucose and glycosylated hemoglobin are significantly associated with macrosomia in pregnant women with GDM [27–29]. Maternal hyperglycemia may cause morphological changes in the placentas of pregnant women. The increase in placental angiogenesis and chorionic branches promotes the transport of glucose to the fetus. Fetal hyperglycemia leads to a compensatory hyperinsulinemic state. A large amount of glucose is metabolized in the fetus, resulting in increased fat and protein storage, which can lead to macrosomia [30, 31]. Moreover, high TG in the third trimester and a high AFI were also independent risk factors for macrosomia [32, 33]. The results of these studies are all similar to those of our study. Therefore, we must strengthen health management during pregnancy and regularly monitor blood sugar and lipids to prevent the occurrence of macrosomia and improve maternal and pediatric outcomes.

In the past, some scholars have developed new methods to predict macrosomia, but they have not been widely used. Liuyu Wu [34] used the Bayes discriminant analysis method combined with maternal examination information to explore a simple model for predicting macrosomia, but this model was not accurate enough and had low clinical practicability. Rongrong Dong [14] used a machine learning method to predict macrosomia. Although this method can improve the accuracy of macrosomia prediction to a certain extent, its sample size is small, and its generalization is poor [15]. Mazouni [35] developed a nomogram model that combines clinical and ultrasound variables to predict macrosomia, and the model has good discrimination and correction. However, it only applies to Europeans and Africans, not a Chinese population. Based on carnitine-related metabolic variables, Man Sun [36] developed a nomogram model for predicting macrosomia in pregnant women with GDM. However, carnitine metabolism is not a routine prenatal examination item, and the use of such a nomogram may increase the economic burden of pregnant women. In 2020, Yanan Xu [37] constructed a prediction model for the risk of macrosomia in pregnant women with GDM in China based on the indexes of routine obstetric examination. However, the sample size of this study was small, and the included predictors were limited. In addition, the model was not validated in this study, and the predictive performance of the model is unknown. In our study, we reviewed a large number of medical records of pregnant women with GDM in China and combined clinical data with ultrasound variables to construct a nomogram model and verified it. The results show that our nomogram model has accurate prediction ability and discrimination, indicating that this nomogram model can accurately predict the risk of macrosomia among pregnant women with GDM in China and has good generalizability. The nomogram model can transform the tedious regression equation into a visual and readable graph, which is convenient and fast for practical applications [38]. This model is conducive to the dynamic evaluation of pregnant women by medical staff according to the different state levels of each single index in the model. The nomogram can help medical staff and pregnant women choose a reasonable mode of delivery and prepare for the delivery and nursing of macrosomia in advance to achieve the ultimate goal of improving pregnancy outcomes.

When using this nomogram model, the medical staff can obtain the point value of each item according to the maternal examination indexes of pregnant women with GDM and sum up all of the points to obtain the total. Then, the risk of macrosomia in pregnant women with GDM can be determined by finding the corresponding point on the total points axis and making a vertical line downward to the risk axis. For example, a pregnant woman with GDM with a prepregnancy BMI of 20 kg/m^2^ (point = 10), weight gain during pregnancy of 20 kg (point = 20), a FPG in the 75 g OGTT of 6 mmol/L (point = 22), TG at 28–32 weeks of gestation of 6 mmol/L (point = 10), BPD at the last prenatal examination of 9 cm (point = 62.5), AFI at the last prenatal examination of 14 cm (point = 13), has a score of 10 + 21 + 22 + 10 + 62.5 + 13 = 138.5, corresponding to a risk of macrosomia is 0.2. This pregnant woman is considered to have a low risk of macrosomia.

### Limitations of the study

This study had some limitations. First, this was a single-center retrospective study, and the generalizability of the results was limited. In the future, a multicenter prospective large-sample study should be designed to include a higher sample size and additional related factors to improve the accuracy of model prediction. Second, due to the retrospective nature of this study, we could not accurately obtain data on the disease treatment of pregnant women with GDM, though such treatment may have had a certain impact on the birth weight of the fetus. Third, this result is applicable only to a Chinese population because our nomogram was constructed using the medical records of pregnant women with GDM in China. Considering the differences between different races and different countries, whether this nomogram model is applicable to populations in other countries remains to be verified.

## Conclusions

In summary, our nomogram model had good differentiation and accuracy, which could help accurately predict the risk of macrosomia and provide a reference for targeted intervention measures. Converting the nomogram into a corresponding software tool is necessary for clinical application.

## Data Availability

The datasets generated and/or analysed during the current study are not publicly available due to limitations of ethical approval involving the patient data and anonymity but are available from the corresponding author on reasonable request.

## Abbreviations

GDM: Gestation diabetes mellitus
OGTT: Oral glucose tolerance test
ROC: Receiver operating characteristic curve
AUC: Area under the curve
BMI: Body mass index
FPG: Fasting plasma glucose
TG: Triglyceride
TC: Cholesterol
S/D: Umbilical artery blood velocity
PI: Pulsatility index
RI: Resistance index
BPD: Biparietal diameter
HC: Head circumference
FL: Femur length
AFI: Amniotic fluid index
CI: Confidence interval
